# Isolation and Characterization of a Novel Cold-Adapted Esterase, MtEst45, from *Microbulbifer thermotolerans* DAU221

**DOI:** 10.3389/fmicb.2016.00218

**Published:** 2016-03-02

**Authors:** Yong-Suk Lee

**Affiliations:** Department of Biotechnology, Dong-A UniversityBusan, South Korea

**Keywords:** bacterial lipolytic enzyme, cold-adapted enzyme, esterase, *Microbulbifer thermotolerans*, DAU221

## Abstract

A novel esterase, MtEst45, was isolated from a fosmid genomic library of *Microbulbifer thermotolerans* DAU221. The encoding gene is predicted to have a mass of 45,564 Da and encodes 495 amino acids, excluding a 21 amino acid signal peptide. MtEst45 showed a low amino acid identity (approximately 23–24%) compared with other lipolytic enzymes belonging to Family III, a closely related bacterial lipolytic enzyme family. MtEst45 also showed a conserved GXSXG motif, G_131_IS_133_YG_135_, which was reported as active site of known lipolytic enzymes, and the putative catalytic triad composed of D_237_ and H_265_. Because these mutants of MtEst45, which was S_133_A, D_237_N, and H_265_L, had no activity, these catalytic triad is deemed essential for the enzyme catalysis. MtEst45 was overexpressed in *Escherichia coli* BL21 (DE3) and purified via His-tag affinity chromatography. The optimal pH and temperature of MtEst45 were estimated to be 8.17 and 46.27°C by response surface methodology, respectively. Additionally, MtEst45 was also active between 1 and 15°C. The optimal hydrolysis substrate for MtEst45 among *p*-nitrophenyl esters (C_2_–C_18_) was *p*-nitrophenyl butyrate, and the *K*_*m*_ and *V*_*max*_ values were 0.0998 mM and 550 μmol/min/mg of protein, respectively. MtEst45 was strongly inhibited by Hg^2+^, Zn^2+^, and Cu^2+^ ions; by phenylmethanesulfonyl fluoride; and by β-mercaptoethanol. Ca^2+^ did not affect the enzyme's activity. These biochemical properties, sequence identity, and phylogenetic analysis suggest that MtEst45 represents a novel and valuable bacterial lipolytic enzyme family and is useful for biotechnological applications.

## Introduction

Lipolytic enzymes, primarily esterase and lipases, belong to the α/β hydrolase superfamily of enzymes that catalyze the hydrolysis and synthesis of ester bonds (Holmquist, 2000). Esterases (EC 3.1.1.1) are enzyme that can hydrolyze short-chain fatty esters (≤C_10_), whereas lipases (EC 3.1.1.3) hydrolyze long-chain acylglycerols (≥C_10_) (Verger, 1997). These enzymes are excellent biocatalysts for various reactions, such as esterification and trans-esterification. Furthermore, lipolytic enzymes are widely used in the production of biofuels, foods, detergents, papers, textiles, pharmaceuticals, and chemicals (Fu et al., 2013; Li et al., 2013; Tran et al., 2013; Leis et al., 2015; Vici et al., 2015), in part due to their chemo- and region- selectivity, and their ability to catalyze reactions without cofactors. These properties not only make them an important class of enzymes for biotechnological applications but also an important group of biocatalysts in organic chemistry (Jaeger and Reetz, 2002; Kim et al., 2007).

Bacterial lipolytic enzymes, esterase and lipases, are classified into eight families (I–VIII) based on their sequence identity and biochemical properties: Family I (termed the true lipase family), Family II (also called the GDSL family), Family III, Family IV (also called the hormone-sensitive lipase, HSL family), and Families V–VIII (Arpigny and Jaeger, 1999). Subsequent research led to the discovery of new enzymes that could not be grouped in these eight families. Handrick et al. revealed a novel nPHB depolymerase, PhaZ7, from *Paucimonas lemoignei* which showed significant homology to a lipase of *Bacillus subtilis*, LipB, and assigned it to a new Family IX (Handrick et al., 2001). Levisson et al. identified a new thermostable esterase, EstD, from *Thermotoga maritima* and assigned it to Family X (Levisson et al., 2007). Yoon et al. identified two lipases, LipG (Lee et al., 2006) and LipEH166 (Kim et al., 2009), from a metagenomic library of uncultured environmental samples and assigned them to families XI and XII, respectively. Family XIII was established by the discovery of esterases from *Geobacillus stearothermophilus* (Est30) (Ewis et al., 2004; Liu et al., 2004) and CEGK from *Geobacillus kaustophilus* (Montoro-García et al., 2009). Rao et al. identified a thermostable esterase, EstA3, from *Thermoanaerobacter tengcongensis* and assigned it to Family XIV (Rao et al., 2011). The most recently established bacterial lipolytic family is Family XV, which includes the thermostable esterase EstGtA2 from *Geobacillus thermodenitroficans* (Charbonneau and Beauregard, 2013).

Cold-adapted enzymes exhibit high catalytic activity at low temperatures. Adaptation to cold is largely attributable to a flexible protein structure that easily accommodates substrates at low temperatures with low activation energy (Struvay and Feller, 2012; Lee et al., 2013). Cold-adapted lipolytic enzymes have several properties that are attractive for industrial applications, such as their high catalytic activity at low temperatures and low thermostability. These properties make cold-adapted enzymes well suited to serve as additives in detergents and as biocatalysts for the biotransformation of labile compounds at low temperatures (Joseph et al., 2008). Many cold-adapted lipolytic enzymes have been discovered in recent years (Roh and Villatte, 2008; Heath et al., 2009; Jeon et al., 2009; Kim et al., 2009; Fu et al., 2011, 2013; Hu et al., 2012; Li et al., 2013; Esteban-Torres et al., 2014).

In a previous study, a fosmid library with approximately 40 kb DNA fragments from *Microbulbifer thermotolerans* DAU221 was constructed using the CopyControl Fosmid Library Production Kit (Epicentre, USA). Fosmid clones with lipolytic activity when cultured on LB-tributylin-chloramphenicol agar were named TB1–TB9. The fosmid clone TB3 was discovered to contain a cold-adapted carbohydrate esterase (Lee et al., 2014). In the present study, another fosmid clone with lipolytic activity, TB8, was characterized.

## Materials and methods

### Bacterial strains, chemicals, media, and plasmids

*Microbulbifer thermotolerans* DAU221 was deposited in the Korean Culture Center of Microorganisms (KCCM 43021, 16S rDNA sequence GenBank ID KC571186) and cultured in Marine Broth 2216 (Difco, Detroit, MI, USA). Plasmids pUC118 and pCC1FOS (Epicentre, Madison, USA) were used to construct the genomic library, and pCold I (TaKaRa, Kyoto, Japan) was used as the protein expression vector. *Escherichia coli* (*E. coli*) JM109 and EPI300-T1 were used for cloning, and BL21 (DE3) was used for protein expression. *E. coli* strains were grown at 37°C in Luria-Bertani (LB) broth supplemented with ampicillin (50 μg mL^−1^) or chloramphenicol (12.5 μg mL^−1^) when required. Tributyrin, *p*-nitrophenyl acetate (C_2_), *p*-nitrophenyl butyrate (C_4_), *p*-nitrophenyl caproate (C_6_), *p*-nitrophenyl caprylate (C_8_), *p*-nitrophenyl caprate (C_10_), *p*-nitrophenyl laurate (C_12_), *p*-nitrophenyl myristate (C_14_), *p*-nitrophenyl palmitate (C_16_), and *p*-nitrophenyl stearate (C_18_) were purchased from Sigma-Aldrich (USA).

### Isolation and sequence analysis of the esterase gene

TB8 clone, a fosmid with lipolytic activity identified from the fosmid library of *M. thermotolerans* DAU221, was partially digested with *Pst*I and then ligated into pUC118 digested with *Pst*I and calf intestinal alkaline phosphatase (CIAP). *E. coli* JM109 was transformed with the ligation mixture using the Hanahan method (Hanahan, 1983). Subclones of TB8 were incubated on LB agar plates containing a tributyrin emulsion (10 mM CaCl_2_, 20 mM NaCl, and 5% gum arabic solution) and ampicillin for 5 days at 37°C (Kim et al., 2008). A positive clone, TB8P1, showed a clear zone surrounding the colony. The recombinant plasmid from this clone, pTB8P1, was partially digested with *Hin*dIII and ligated into the pUC118/*Hin*dIII/CIAP. The ligation mixture was transformed into *E. coli* JM109, which was then incubated on LB-tributyrin-ampicillin agar for 5 days at 37°C. A positive clone, TB8P1H1, showed a halo around the colony and was selected for sequencing. Sequence similarity searches were performed using BLAST (National Center for Biotechnology Information, NCBI). The signal peptide sequence was analyzed using the SignalP 3.0 server (http://www.cbs.dtu.dk/services/SignalP) (Peterson et al., 2011). Amino acid sequencing alignments of the identified bacterial lipolytic enzyme and homologous proteins were analyzed using the ClustalW program (Thompson et al., 1994). A phylogenetic tree was constructed using the neighbor-joining method with 1000 bootstrap replicates using the Molecular Evolutionary Genetics Analysis software (MEGA version 6.0.5) (Tamura et al., 2013). Three dimensional (3D) structure of MtEst45 was modeled using the Phyre^2^ server (Protein Homology/analygY Recognition Engine Ver. 2.0) (http://www.sbg.bio.ic.ac.uk/phyre/) (Kelly et al., 2015). The 3D structure was visualized using a PyMOL software (http://www.pymol.org) (DeLano, 2002).

### Expression and purification of esterase

The esterase gene from DAU221 was amplified without its signal peptide (the first 21 amino acids) and its stop codon using chromosomal DNA of *M. thermotolerans* as a template and the following two primers: 5′-GCGCACAGC*GAATTC*GCGCCGCGCTTTATT-3′ (forward, *Eco*RI restriction enzyme site in italics and underlined) and 5′-GTCCGGGAC*GTCGAC*TCACAGTTGCGGTAT-3′ (reverse, *Sal*I restriction enzyme site in italics and underlined). The PCR product was double-digested by *Eco*RI and *Sal*I restriction enzymes and ligated into the corresponding sites in the pCold I vector. The recombinant plasmid pCold-MtEst45-SP (designed to introduce a hexa-histidine tag at the N-terminus of the enzyme to facilitate purification) was transformed into *E. coli* JM109 and BL21 (DE3). A single colony of *E. coli* BL21 (DE3)/pCold-MtEst45-SP was inoculated into 10 mL LB medium containing ampicillin and incubated overnight at 37°C with shaking. When the optical density at 600 nm reached 0.4–0.5, protein expression was induced with 0.2 mM isopropyl-β-D-thiogalactoside (IPTG) for 24 h at 15°C. Cells were harvested via centrifugation at 5590 × g force for 15 min at 4°C and the pellet was re-suspended in binding buffer (20 mM sodium phosphate buffer [pH 8.0], 0.5 M NaCl, and 5 mM imidazole). The cells were disrupted via sonication on ice (pulse-on 30 s, pulse-off 30 s, 5 times), and the supernatant was collected via centrifugation at 16,000 × g force for 30 min at 4°C. The clear supernatant was loaded on to a HisTrap HP column (Amersharm Bioscience) that had been equilibrated with binding buffer, and the bound protein was eluted with elution buffer (20 mM sodium phosphate buffer [pH 8.0], 0.5 M NaCl, and 0.5 M imidazole) at a flow rate of 1 mL min^−1^. The eluted fractions were dialyzed overnight against 20 mM Tris-HCl buffer (pH 8.17) and concentrated using Amicon Ultra-4 spin columns (Millipore, Bedford, MA, USA). The protein concentrations of the enzyme preparations were determined following the method described by Bradford (1976) with bovine serum albumin as a standard (0.2–1 mg mL^−1^). Sodium dodecyl sulfate-polyacrylamide gel electrophoresis (SDS-PAGE) was performed using the Laemmli method (Laemmli, 1970).

### Enzyme assay

Esterase activity was determined by measuring the release of *p*-nitrophenol during the enzymatic hydrolysis of different *p*-nitrophenyl esters (Sigma-Aldrich). The release of *p*-nitrophenol was measured at 405 nm using an Ultrospec 2000 pro UV/visible spectrophotometer (Amersham Bioscience). In a total volume of 1.0 mL, esterase was incubated with 0.25 mM *p*-nitrophenyl butyrate (C_4_) as a substrate in 20 mM Tris-HCl buffer (pH 8.17) at 46.27°C for 30 min. One unit of enzyme activity was defined as the amount needed to release 1 μmol *p*-nitrophenol per min.

### Characterization of the enzyme

An optimum pH for enzyme activity was determined over a pH range from 3.0 to 9.0. The reaction buffers (at a concentration of 20 mM) used were citrate buffer for the 3.0–6.0 pH range, sodium phosphate buffer for the 6.0–8.0 pH range, and Tris-HCl buffer for the 7.5–9.0 pH range.

To determine the optimal temperature for enzyme activity, the hydrolytic activity of esterase was measured at temperatures between 1 and 70°C under standard conditions (20 mM Tris-HCl buffer [pH 8.17]). Esterase thermostability was determined by pre-incubating the esterase at 25–70°C for up to 3 h before conducting the activity tests described above.

To optimize the pH and temperature condition for enzyme activity, response surface methodology (RSM) was explored. The central composite design consisting of 13 experimental runs with 5 replicates at the center point was used to optimize the independent variables which significantly influenced the enzyme activity. Based above experiments, two critical factors and their optimal ranges were selected in this experiment: pH (*X*_*1*_, 7.29–8.71) and temperature (*X*_2_, 30.86–59.14°C; Table 1). Empirical model equation (Equation 1) was created that correlated the relationship between the independent variables and the dependent variable by the statistical software Design-Expert (Version 8.0.7.1; Stat-Ease Inc., Minneapolis, USA).

(1) $$\begin{array}{r} \text{Y}={\text{β}}_{0}+\sum \beta_{\text{i}}{\text{X}}_{\text{i}}+\sum \beta_{\text{ij}}{\text{X}}_{\text{i}}{\text{X}}_{\text{j}}+\sum \beta_{\text{ii}}{\text{X}}_{\text{i}}^{2} \end{array}$$

where Y is the predicted response, X_i_ and X_j_ are variables, β_0_ is the constant, β_*i*_ is the linear coefficient, β_ij_ is the interaction coefficient, and β_ii_ is the quadratic coefficient.

**Table 1:** **The central-composite design for optimizing MtEst45 activity**.

	***X_*1*_***	***X_*2*_***	***Y***
1	45.00	8.00	3.467
2	35.00	8.50	2.399
3	45.00	8.00	3.549
4	45.00	8.00	3.449
5	30.86	8.00	1.780
6	45.00	8.00	3.482
7	45.00	7.29	2.544
8	55.00	7.50	2.713
9	35.00	7.50	1.482
10	59.00	8.00	2.254
11	45.00	8.00	3.497
12	55.00	8.50	2.692
13	45.00	8.71	3.289

*X_1_, Temperature (°C); X_2_, pH; Y, p-nitrophenol (μg)*.

Various additives were used to establish their influence on the MtEst45 activity. To determine the effects of metal ions on enzymatic activity, the enzyme was pre-incubated with BaCl_2_, CaCl_2_, CsCl, CuCl_2_, FeCl_2_, HgCl_2_, KCl, LiCl, NiCl_2_, MgCl_2_, MnCl_2_, or ZnCl_2_ at final concentrations of 1, 5, and 10 mM for 24 h at 4°C. NaCl (0.5, 1, 2, 3, and 4 mM) was pre-incubated with the enzyme for 12 h at 4°C. Furthermore, the enzyme was pre-incubated with 1, 5, or 10 mM ethylenediaminetetraacetic acid (EDTA) for 12 h at 4°C as well as diethylpyrocarbonate (DEPC) and β-mercaptoethanol, both for 6 h at 4°C.

Phenylmethanesulfonyl fluoride (PMSF) on the esterase activity was also assessed. PMSF was pre-incubated with the enzyme for 10 min at 4°C before the enzyme was assayed. This short time period was used because the half-life of aqueous PMSF at pH 8.17 is 35 min, which renders it an unstable material in the presence of water (James, 1978). Finally, the enzyme was assayed for esterase activity in the presence of 10% (vol/vol) dimethyl sulfoxide (DMSO).

The enzyme's substrate specificity for *p*-nitrophenyl esters with different lengths from C_2_ to C_18_ was determined at 46.27°C and pH 8.17. All *p*-nitrophenyl esters were prepared by dissolving the substrates in ethanol.

All of the experiments were performed in triplecates under standard assay conditions. The esterase activity measured without additives was set to represent 100% activity.

To determine the *K*_*m*_ and *V*_*max*_ values, varying concentrations of *p*-nitrophenyl butyrate (C_4_) substrates (0.1–1 mM) were hydrolyzed using esterase under the standard assay conditions. Kinetic parameters were obtained from Lineweaver-Burk plots against the different substrate concentrations using the SWIFT II Applications software (Amersham Bioscience).

### Site-directed mutagenesis

Mutations were generated using the QuikChange Site-directed Mutagenesis Kit (Stratagene, San Diego, CA, USA) with the following primers:

S_133_A-F (5′-GATGGCCGGTATTGCTTACGGCGCCGGAAT-3′),
S_133_A-R (5′-ATTCCGGCGCCGTAAGCAATACCGGCCATC-3′),
D_237_N-F (5′-CGCCAACTACCTGTTCAAACCCCAACAGCACC-3′),
D_237_N-R (5′-GGTGCTGTTGGGTTTGAACAGGTAGTTGGCG-3′),
H_265_L-F (5′-TTAACCCGGGAACCCTCGCGGTGACCGAGA-3′),
H_265_L-R (5′-TCTCGGTCACCGCGAGGGTTCCCGGGTTAA-3′) (mutations are underlined).

Complementary primers with the wild-type gene bearing the nucleotides to be changed were used for PCR. The PCR mixtures were treated with *Dpn*I to digest the methylated template DNA. The mutation sites were confirmed via DNA sequencing. Plasmids were transformed into *E. coli* BL21 (DE3) cells for protein expression.

### Nucleotide sequence accession numbers

The nucleotide sequence reported in this study has been deposited in GenBank under the accession number KM362852.

## Results

### Isolation and sequence analysis of MtEst45

The fosmid library was screened for lipolytic activity on LB-tributyrin agar media. Clones positive for tributyrin degradation were isolated and named TB1–TB9. The lipolytic-active TB8 clone was selected, and a subcloned library of TB8 was constructed via consecutive enzyme digestions with *Pst*I and *Hin*dIII. TB8P1H1 exhibited lipolytic activity and contained a 5509 kb DNA insert. An open reading frame (ORF) analysis revealed two ORFs encoding proteins of over 300 amino acids from amongst six putative ORFs. The protein encoded by ORF1 shared high sequence identity with an acyl esterase from *Microbulbifer agarilyticus* (WP_010133327). The protein encoded by ORF2 had 85% sequence identity (354/416) with TldD protein from *Microbulbufer* sp. ZGT114 (KUJ83017).

In ORF1, the esterase gene *mtest45* from *M. thermotolerans* DAU221 begins with an ATG at nucleotide 2089 and ends with a TGA at nucleotide 3639. A putative ribosome-binding site, 5′-AGAGAGA-3′, is located six bp upstream from the initiation codon ATG. Thus, the DAU221 *mtest45* gene is 1551 bp and encodes 516 aa with deduced molecular mass of 56,742 Da and an isoelectric point of 5.65. The signal peptide sequence was analyzed using the SignalP server, and the most likely cleavage sites were presumed to be between Ala_21_ and Ala_22_. Hence, the mature protein was predicted to contain 495 amino acids with a deduced molecular mass of 45,564 Da and an isoelectric point of 5.48.

**Figure 1:**
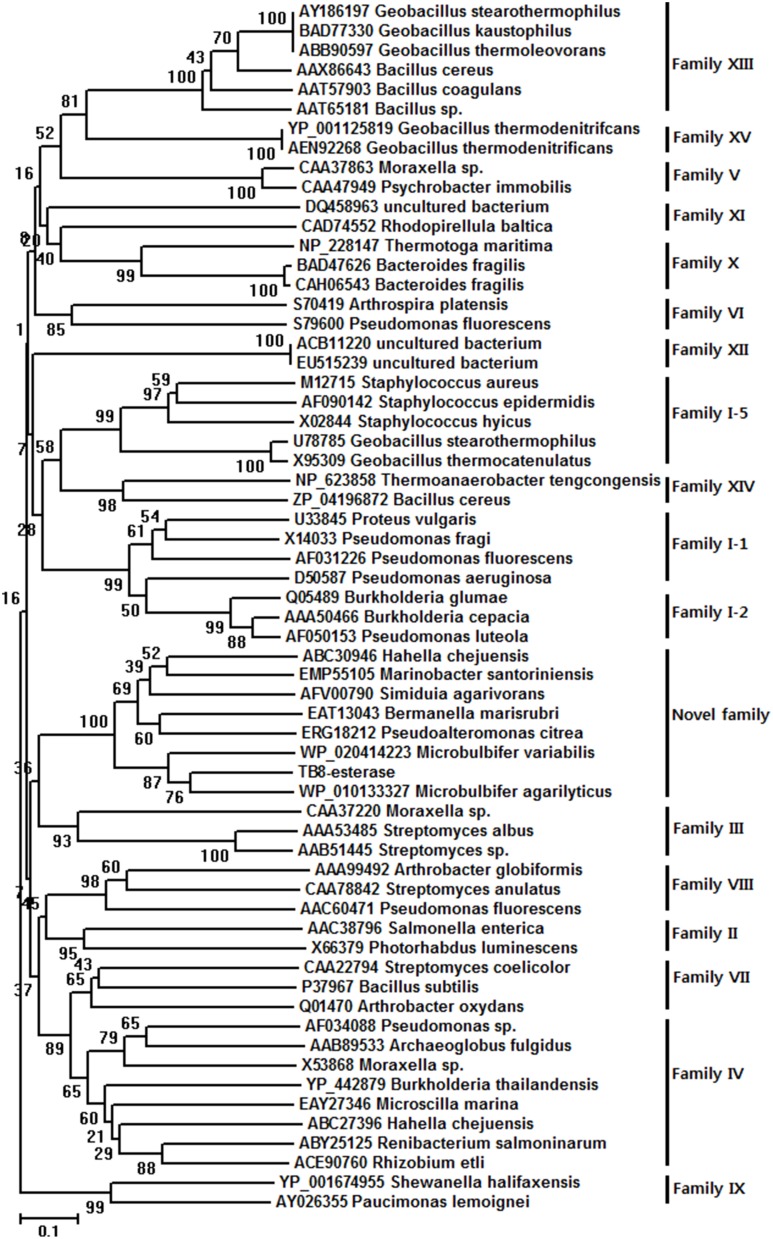
**Unrooted neighbor-joining phylogenetic tree of MtEst45(TB8-esterase) and related lipolytic enzymes**. The amino acid sequences of the bacterial lipolytic enzymes were referred to the previously classified 15 bacterial lipolytic families. Sequence alignment was performed using CLUSTAL W and the tree was created with the MEGA program version 6.0.5.

The deduced amino acid sequence of the putative esterase gene was compared with known esterase and lipase amino acid sequences available from GenBank (NCBI databank). The MeEst45 shared 76% sequence identity (384/502) with an acyl esterase from *Microbulbifer agarilyticus* (GenBank Accession No. WP_010133327), 71% identity (364/516) with a hypothetical protein from *Microbulbifer variabilis* (WP_020414223), 42% identity (220/524) with an acyl esterase from *Marinobacter santoriniensis* (EMP55105), 43% identity (214/501) with an acyl esterase from *Hahella chejuensis* (ABC30946), 42% identity (208/494) with an acyl esterase from *Bermanella marisrubri* (EAT1304342% identity (212/500) with an acyl esterase from *Pseudoalteromonas citrea* (ERG18212) (WP_010363576), and 39% identity (194/500) with an acyl esterase from *Simiduia agarivorans* (AFV00790). In order to identify the precise relationship between MeEst45 and other known bacterial lipolytic enzyme, a phylogenetic tree was constructed (Figure 1), based on the 15 bacterial lipolytic hydrolysis families.

**Figure 2:**
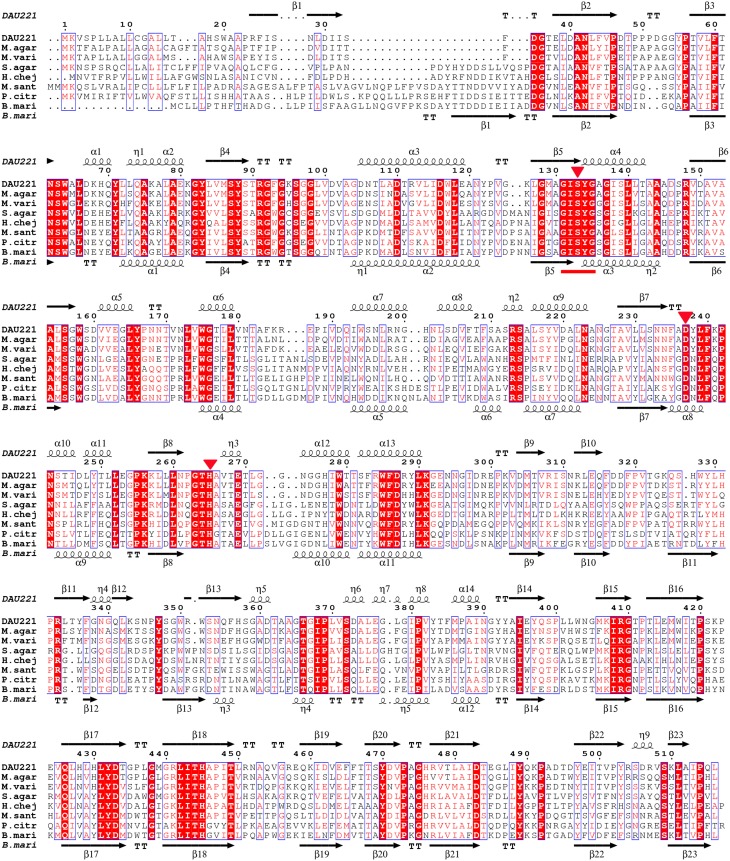
**Alignment of MtEst45 sequence with the novel family of bacterial lipolytic enzymes**. Sequence alignments were performed using the CLUSTAL W and ESPript 3.0 programs. Similar residues are indicated by a clear box and identical residues are indicated by a colored background. The GXSXG motif is underlined in red and the catalytic triad residues is represented by a red inverted-triangle. The secondary structure elements (alpha helix [α], beta sheet [β], random coil [η], and beta turn [T]) are shown above the alignment for MtEst45 and below for B.mari. DAU221, esterase (MtEst45) from *Microbulbifer thermotolerans* DAU221 (KM362852); M.agar, an acyl esterase from *Microbulbifer agarlyticus* (WP_010133327); M.vari, a hypothetical protein from *Microbulbifer variabilis* (WP_020414223); S.agar, an acyl esterase from *Simiduia agarivorans* (AFV00790); H.chej, an acyl esterase from *Hahella chejuensis* (ABC30946); M.sant, an acyl esterase from *Marinobacter santoriniensis* (EMP55105); P.citr, an acyl esterase from *Pseudoalteromonas citrea* (ERG18212); and B.mari, an acyl esterase from *Bermanella marisrubri* (EAT13043).

The multiple sequence alignments of regions with the highest homology are shown in Figure 2. The results indicate that MtEst45 shares the typical serine catalysis penta-peptide GXSXG in protein sequence and also has common catalytic triad, which is consisted of Ser_133_, Asp_237_, and His_265_ (Brenner, 1988; Cygler et al., 1993; Arpigny and Jaeger, 1999).

### Expression and purification of MtEst45

**Figure 3:**
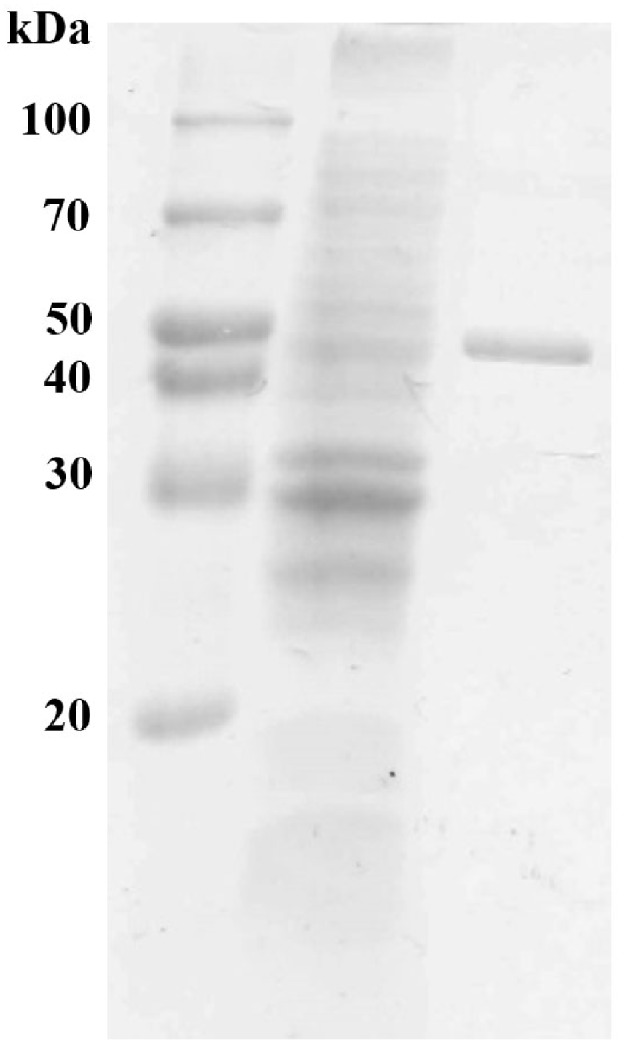
**SDS-PAGE of MtEst45 from ***M. thermotolerans*** DAU221**. Lane 1 represents molecular weight standard proteins, lane 2 represents cell-free extract, and lane 3 shows purified MtEst45 from His-tag affinity chromatography.

The gene sequence encoding the mature form of MtEst45 was amplified without the signal peptide and cloned into the pCold I vector for the enzyme expression and purification. The resulting expression plasmid pCold-MtEst45-SP contained a six histidine tag at the N-terminus of MtEst45 and was transformed into *E. coli* BL21 (DE3) for protein expression. *E. coli* BL21 (DE3) harboring pCold-MtEst45-SP was induced overnight with 0.2 mM IPTG at 15°C. The heterologously expressed MtEst45 protein was purified using His-tag affinity chromatography. SDS-PAGE analysis of the purified protein revealed a molecular weight (MW) of approximately 45 kDa under denaturing conditions (Figure 3).

### The effects of temperature and pH on MtEst45 activity

**Figure 4:**
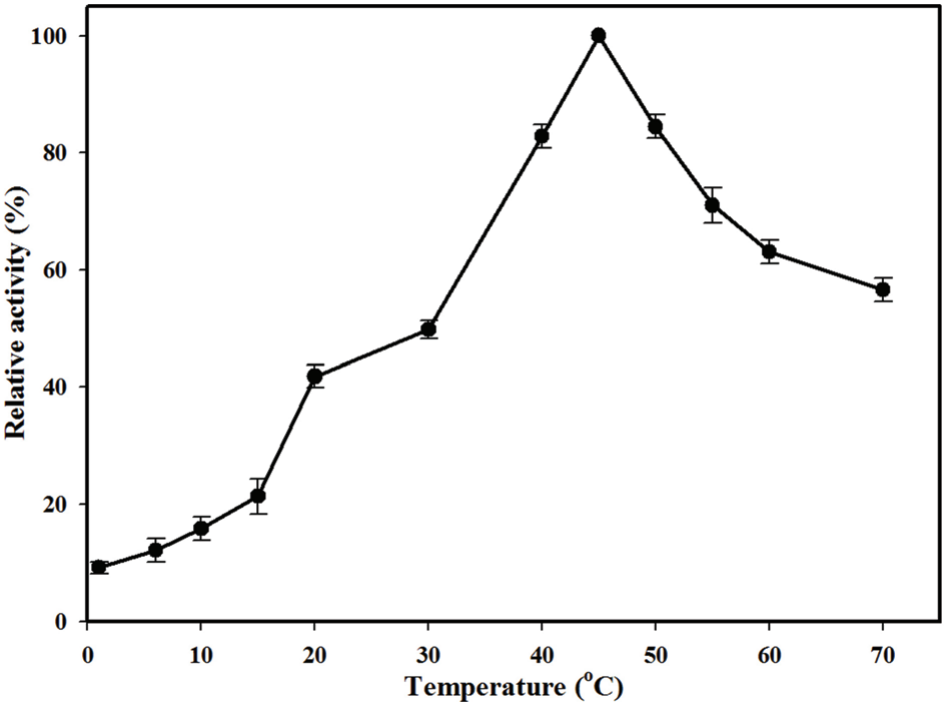
**Effects of temperature on the activity of MtEst45**. Temperatures range from 1 to 70°C. Each reaction sample was compared with the standard assay control without any additive. The means of the relative values (*n* = 3) and standard deviations are shown.

The effect of pH on MtEst45 activity was determined using *p*-nitrophenyl butyrate (C_4_) as a substrate with a broad pH range from 3.0 to 9.0 (data not shown). The MtEst45 activity was maximal at pH 8.0. In sodium phosphate buffer, MtEst45 retained over 70% activity from pH 7.0 to 8.0 but decreased drastically at pH 6.0 to 6.5. In citrate buffer, MtEst45 maintained approximately 50% of its maximal activity from pH 3.0 to 6.0. The optimal temperature was determined to occupy a range from 1 to 70°C (Figure 4). MtEst45 activity was maximal at 45°C in 20 mM Tris-HCl buffer, pH 8.0, >80% at 40 and 50°C, and >50% at 55–70°C, respectively. Notably, MtEst45 enzyme activity was maintained between 1 and 15°C.

**Figure 5:**
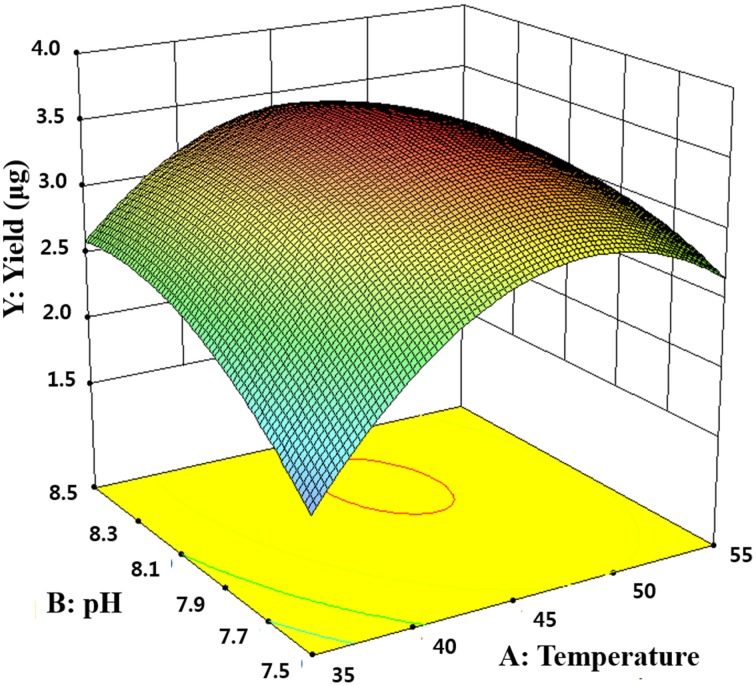
**Response surface plot showing the effects of pH and temperature on MtEst45 activity**.

The 13-runs, for two independent variables including pH (*X*_*1*_) and temperature (*X*_*2*_) were manipulated and optimized for MtEst45 activity. The results of the quadratic polynomial model fitting in the term of analysis of variance (ANOVA) were given in Table 2. A quadratic polynomial function was fit to the experimental values, resulting in the following regression equation (Equation 2):

(2) $$\begin{array}{r} \text{Y}=3.49+0.27X_{\text{1}}+0.24X_{\text{2}}-0.23X_{1}X_{\text{2}}-0.77X_{\text{1}}^{\text{2}}-0.32X_{\text{2}}^{\text{2}} \end{array}$$

where Y is the predicted *p*-nitrophenol yield (μg). The model F-value of 57.04 implies that the model is significant. The *P* < 0.05 suggests that the model is considered to be statistically significant at >95% confidence level. The large value of the regression coefficient (*R*^2^ = 0.9760) indicates that only 2.4% of the total variations are no explained by the model. The value of the adjusted determination coefficient (Adj *R*^2^ = 0.9589) is also very high to advocate for a high significance of the model. The low coefficient of variation (CV = 5.06%) also reveals that the model was accurate and reliable (Lee et al., 2015). A three-dimensional response surface was then plotted to illustrate the effects of pH and temperature (Figure 5). The model predicts a maximum enzyme activity of MtEst45 was observed to be 8.17 (pH; *X*_*1*_) and 46.27 (°C; *X*_*2*_), respectively.

**Table 2:** **Analysis of variance (ANOVA) for the fitted quadratic polynomial model for MtEst45 activity**.

**Source**	**D.F**.	**S.S**.	**M.S**.	***F*-value**	*P*-value^*^
Model	5	5.79	1.16	57.04	<0.0001
*X*_*1*_	1	0.60	0.60	29.63	0.0010
*X*_*2*_	1	0.48	0048	23.39	0.0019
*X*_*1*_X_*2*_	1	0.22	0.22	10.83	0.0133
$X_{1}^{2}$	1	4.15	4.15	204.22	<0.001
$X_{2}^{2}$	1	0.72	0.72	35.61	0.006
Lack of fit	3	0.14	0.45	31.37	0.0031
Error	4	0.005797	0.00449	-	-
Total	12	5.94	-	-	-

*R^2^, 0.9760; D.F., refers to degrees of freedom; S.S., refers to sum of sequences; M.S. refers to mean square*.

* *P-value less than 0.05 indicates the model terms are significant*.

**Figure 6:**
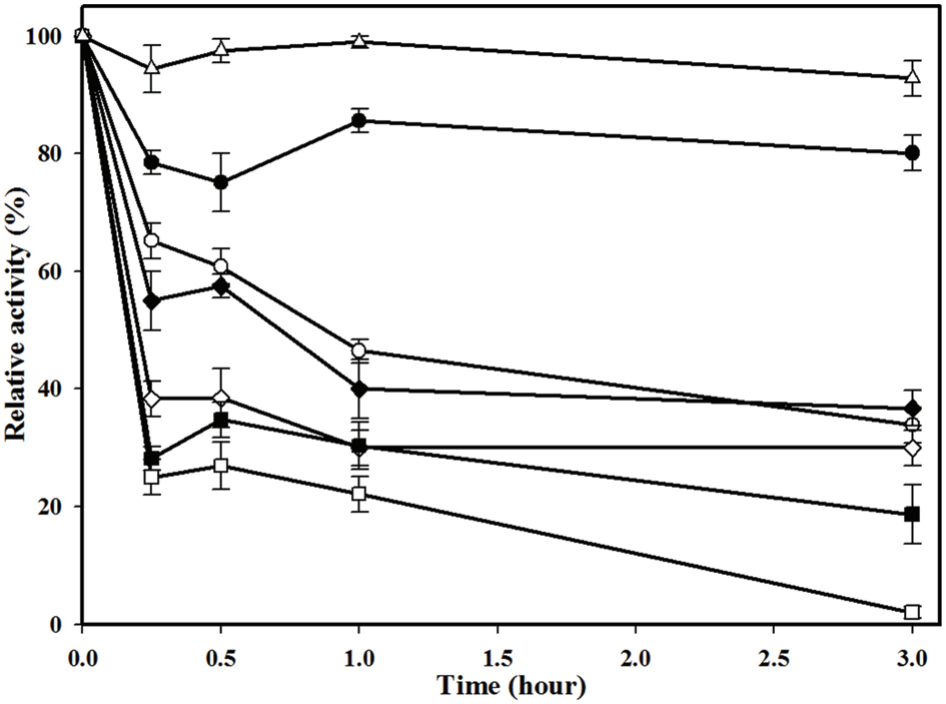
**Thermostability of MtEst1**. Enzyme was pre-incubated for various times (0.25, 0.5, 1, and 3 h) at 25°C (white triangle), 30°C (black circle), 40°C (white circle), 45°C (black diamond), 50°C (white diamond), 60°C (black quadrangle), and 70°C (white quadrangle). Each reaction sample was compared with the standard assay control without any additive. The means of the relative values (*n* = 3) and standard deviations are shown.

The thermostability of MtEst45 was also determined via residual activity analysis at regular intervals after pre-incubating the enzyme for up to 3 h at various temperatures (25–70°C; Figure 6). MtEst45 was stable after incubation for 3 h at 25 and 30°C with residual activities of greater than 90 and 75%, respectively. At 40 and 45°C, MtEst45 maintained approximately 55–65% and >30% of its maximal activity after 15 min and 3 h of incubation, respectively. At 50 and 60°C, MtEst45 maintained approximately 30–40% of its maximal activity after 15 min of incubation and 30 and 18% of its maximal activity at 50 and 60°C, respectively, after 3 h of incubation. At 70°C, MtEst45 maintained 17% of its maximal activity after 15 min of incubation but lost its activity after 3 h of incubation.

### The effects of metal ions and additives on MtEst45 activity

**Figure 7:**
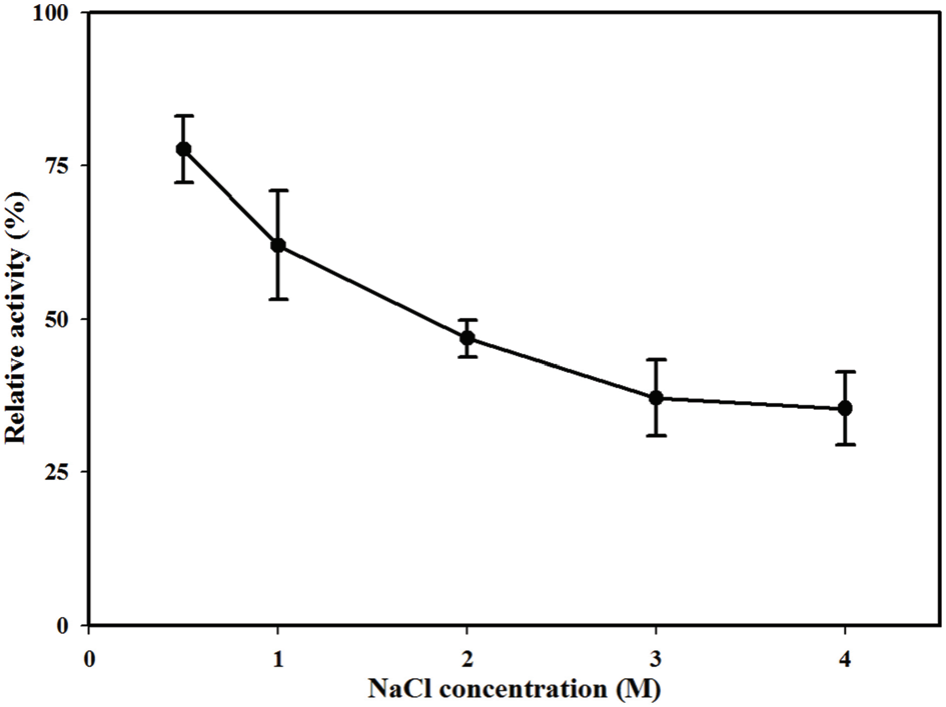
**Effect of NaCl concentration on MtEst1 activity**. The means of the relative values (*n* = 3) and standard deviations are shown.

The effects of metal ions and additives on MtEst45 activity are depicted in Table 3. Ba^2+^, Ca^2+^, Cs^2+^, K^+^, and Li^+^ did not show any drastic effects on enzyme activity at the concentrations tested (1, 5, and 10 mM) with approximately 20–40% reduced enzyme activity. Co^2+^ reduced enzyme activity by approximately 25–30% at 1 and 5 mM but by 60% at 10 mM. Mg^2+^ and Mn^2+^ reduced enzyme activity by 25–35% at 1 mM; however, concentrations of 5 and 10 mM reduced enzyme activity by 60 and 70%, respectively. All Hg^2+^ and Zn^2+^ concentrations, 5 and 10 mM Cu^2+^, and 10 mM Ni^+^ had a strong negative effect on enzyme activity, suggesting that these metal ions could be potent inhibitors. EDTA had no significant effect on enzyme activity, only reducing it by 5–15%. DEPC reduced enzyme activity by approximately 25% at 1 mM and 40% at 5 mM but reduced it by 60% at 10 mM. MtEst45 activity was completely blocked by 10 mM β-mercaptoethanol as well as 5 and 10 mM PMSF. At low NaCl concentrations (0.5 and 1 M), enzyme activity could be reduced by 25 and 45%, respectively. When MtEst45 was pre-incubated with 2, 3, and 4 M NaCl, MtEst45 showed approximately 45–35% of enzyme activity compared with the standard enzyme assay conditions (Figure 7).

**Table 3:** **Effect of different metal ions and additives on MtEst45 activity**.

**Compounds**	**Relative activity (%)**^a^		
	**1 mM^b^**	**5 Mm**	**10 mM**
Control	100	100	100
K^+^	82.4 ± 1.2	83.7 ± 1.3	75 ± 1.6
Ba^2+^	80.9 ± 1.3	74.1 ± 0.9	60.3 ± 0.6
Li^+^	74.6 ± 1.6	75.5 ± 0.3	73.2 ± 1.3
Cs^2+^	66.0 ± 3.6	69.0 ± 1.4	66.7 ± 0.6
Ca^2+^	76.5 ± 1.5	77.0 ± 0.3	59.4 ± 0.5
Co^2+^	75.2 ± 1.4	67.2 ± 1.4	42.3 ± 1.1
Mn^2+^	78.8 ± 3.3	42.4 ± 0.6	30.5 ± 0.3
Mg^2+^	62.6 ± 1.2	38.6 ± 0.5	32.2 ± 1.2
Ni^+^	51.8 ± 0.6	47.7 ± 0.8	5.9 ± 0.32
Cu^2+^	36.7 ± 0.2	N.D.^c^	N.D.
Zn^2+^	N.D.	N.D.	N.D.
Hg^2+^	N.D.	N.D.	N.D.
EDTA	94.8 ± 0.4	84.9 ± 0.6	87.7 ± 1.0
DEPC	77.0 ± 0.8	62.0 ± 1.2	37.1 ± 0.8
ß-mercaptoethanol	53.5 ± 0.4	40.5 ± 1.5	9.4 ± 0.6
PMSF	20.5 ± 0.8	N.D.	N.D.

*Values represent the mean of three replicates*.

a *The activity is expressed as a percentage of the activity of the untreated control*.

b *Concentration in pre-incubation mixture*.

c *N.D., Not Detected*.

### Substrate specificity and kinetic parameters of MtEst45

To confirm the biochemical characterization of MtEst45, the substrate specificity of the purified protein was analyzed using *p*-nitrophenyl esters from *p*-nitrophenyl acetate (C_2_) to *p*-nitrophenyl stearate (C_18_) (data not shown). The highest specific activity of MtEst45 was found for *p*-nitrophenyl butyrate (C_4_). Minimal activity was found toward short chain acetate (C_2_) and caproate (C_6_). Consequently, the enzyme can be classified as an esterase rather than a lipase (Arpigny and Jaeger, 1999). *K*_*m*_ and *V*_*max*_ values of MtEst45 were calculated from Lineweaver-Burk plots against various *p*-nitrophenyl butyrate (C_4_) concentrations and were 0.0998 mM and 550 μmol/min/mg of protein, respectively.

### Structural modeling

**Figure 8:**
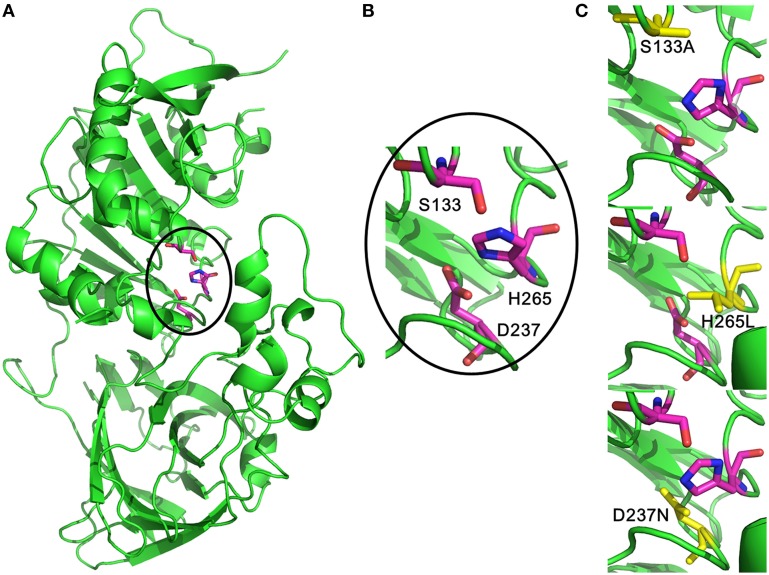
**Three-dimensional structural feature of MtEst45. (A)** The cartoon representation of MtEst45 model (green). The carbon atoms for the catalytic triad residues are shown in magentas. **(B)** The detailed picture of the catalytic triad residues: S_133_, D_237_, and H_265_. **(C)** Structural picture of the S_133_A, D_237_N, and H_265_L mutants. The substituted amino acid residues are shown in yellow.

The 3D models of MtEST45 and mutants were made using the Phyre^2^ server (Figure 8). In the obtained model, Ser_133_, Asp_237_, and His_265_ were indeed located in close proximity, most likely representing the actual active site. To confirm the predictions of the catalytic triad, these residues were substituted by site-directed mutagenesis. No activity was found in Ser_133_, Asp_237_, and His_265_ mutants, showing that these amino acids are likely to be essential for the enzyme catalysis.

## Discussion

Here the physiological and biochemical aspects of an esterase from *M. thermotolerans* DAU221 were characterized. The *M. thermotolerans* DAU221 strain was isolated on the eastern coast of the Republic of Korea (Lee et al., 2014). The two hydrolases, a carbohydrate esterase (Lee et al., 2014) and a maltotriose-producing α-amylase (Lee et al., 2015), from marine bacterium *M. thermotolerans* DAU221 were discovered. Marine environments are rich in various lipids, such as bacterial membranes are of great importance given environmental stresses of extreme temperature and pressure. Thus, marine habitats are potentially rich in novel genetic sources of lipolytic enzymes (Hu et al., 2010; Fu et al., 2011).

**Figure 9:**
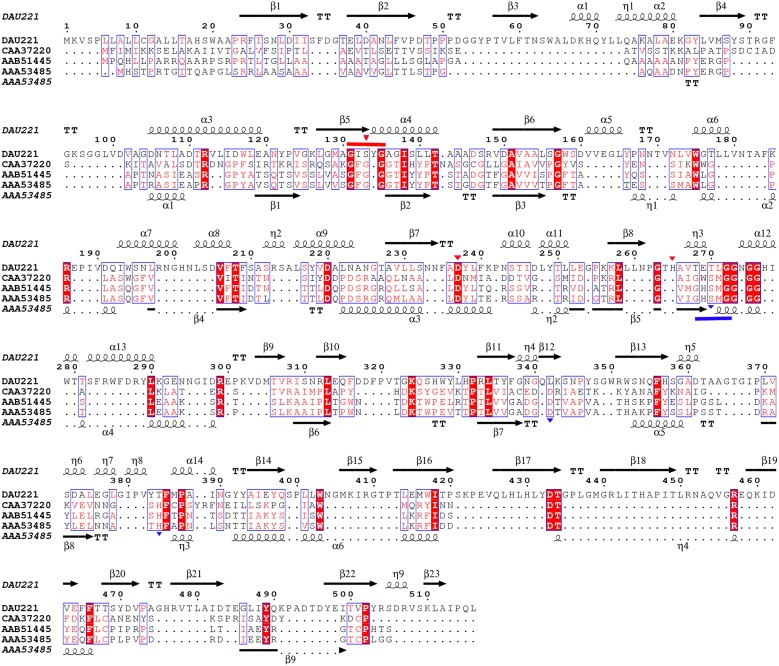
**Alignment of MtEst45 sequence with Family III of the bacterial lipolytic enzymes**. Sequence alignments were performed using the CLUSTAL W and ESPript 3.0 programs. The GXSXG motif of MtEst45 (red) and the Family III lipolytic enzymes (blue) were lined and the catalytic triad were represented by inverted-triangle, respectively. DAU221, esterase (MtEst45) from *Microbulbifer thermotolerans* DAU221 (KM362852); CAA37220, putative lipase from *Moraxella* sp.; triacylglycerol acylhydrolase from *Streptomyces* sp.; and AAA53485, lipase from *Streptomyces albus*. The secondary structure elements (alpha helix [α], beta sheet [β], random coil [η], and beta turn [T]) are shown above the alignment for MtEst45 and below for AA53485 (*Streptomyces albus*).

Phylogenetic analysis revealed that MtEst45 from *M. thermotolerans* DAU221 and its homologs were closely related to Family III lipolytic enzymes, and branch as a sister group of Family III in the tree. Family III lipolytic enzymes display the canonical fold of α/β hydrolases and contain a typical catalytic triad for serine hydrolases (Arpigny and Jaeger, 1999). Family III was primarily characterized by Feller et al. (1990), and a 3D structure of *Streptomyces exfoliates* M11 lipase, which belongs to Family III, was reported (Cruz et al., 1994). MtEst45 showed a low amino acid sequence identity and similarity to esterases/lipases of the Family III group (Figure 9): 24% identity (35/145) and 40% similarity (59/145) with lipase from *Moraxella* sp. TA144 (CAA37220) (Feller et al., 1990), 24% identity (26/109) and 39% similarity (43/109) with lipase from *Streptomyces albus* G (AAA53485) (Cruz et al., 1994), 23% identity (9/39) and 48% similarity (19/39) with lipase from *Streptomyces* sp. (AAB51445) (Pérez et al., 1993), and 23% identity (3/39) and 48% similarity (19/39) with lipase from *Streptomyces exfoliates* M11 (PDB 1JFR) (Wei et al., 1998). Based on their sequence identities and enzymatic properties, these enzymes have been classified into a novel family (Figure 2; Arpigny and Jaeger, 1999; Handrick et al., 2001; Lee et al., 2006; Levisson et al., 2007; Kim et al., 2009; Montoro-García et al., 2009; Rao et al., 2011; Charbonneau and Beauregard, 2013).

The optimal activity of MtEst45 was observed at 46.27°C by RSM, whereas 10% of its maximal activity remained at 1°C (0.1959 μmol/min/mg of protein; Figure 4), suggesting cold adaptation. Many cold-adapted lipolytic enzymes have been reported. EstF from a deep-sea metagenomic library showed optimal activity at 35°C and 9% at 0.5°C (Fu et al., 2013), a lipase from *Stenotrophomonas maltophilia* showed optimal activity at 35°C and 57% at 5°C (Li et al., 2013), and an esterase from *Lactobacillus plantarum* showed optimal activity at 20°C and 90% at 5°C (Esteban-Torres et al., 2014). According to previous studies, many cold-adapted enzymes have an overall increased conformation flexibility that appears to be obtained by (i) more Gly residues, (ii) a lower number of Pro and Arg residues, (iii) fewer hydrogen bonds and fewer ion pair interactions, (iv) more Ser and Met residues, (v) reduced hydrophobicity (particularly in internal residues) expressed as a lower (Ile+Leu)/(Ile+Leu+Val) ratio, and (vi) a more optimized surface charge (Smalås et al., 2000; Georlette et al., 2004; Siddiqui and Cavicchioli, 2006; Fu et al., 2013). Thus, the amino acid composition of MtEst45 was analyzed without the signal peptide sequence. MtEst45 contained 45 Gly residues (9.1%), 29 Pro residues (5.9%), 21 Arg residues (4.2%), 32 Ser residues (6.5%), 7 Met residues (1.4%), and 25 Ile residues (5.1%). Gly residues enhance flexibility. Met residues are long, have a high degree of freedom, lack branching, and have no charge or dipole interaction; thus, they are likely to increase flexibility and are a cold-adapted determinant for some enzymes (Smalås et al., 2000; Siddiqui and Cavicchioli, 2006; Fedoy et al., 2007). Furthermore, MtEst45 is characterized by a low Arg/(Arg+Lys) ratio, 0.429 (21/49). Similarly, Lp_2631, cold-active esterase, from *Lactobacillus plantarum* WCFS1 contained 19 Gly residues (7.3%), 15 Pro residues (5.7%), 9 Arg residues (3.4%), 9 Ser residues (3.4%), 6 Met residues (2.3%), 13 Ile residues (5.0%), and a low Arg/(Arg+Lys) ratio, 0.45 (9/20) (Esteban-Torres et al., 2014). However, EstE1, thermophilic esterase, from a metagenomics library contained 23 Gly residues (7.4%), 21 Pro residues (6.8%), 22 Arg residues (7.1%), 22 Ser residues (7.1%), 5 Met residues, 14 Ile (4.5%), and Arg/(Arg+Lys) ratio, 0.619 (13/21) (Zhu et al., 2013). These characteristics of the primary structure provide further evidence indicating that MtEst45 is a cold-adapted esterase.

Metal ions play a role in the maintenance of enzyme stability and active structures (Table 1); in particular, calcium ions (Ca^2+^) are essential to the activities of some esterase. The stimulatory effect of Ca^2+^ may be attributed to better attachment of the enzyme onto the substrate molecule and to the neutralization of fatty acids liberated from the substrate (Lee and Lee, 1990). Ca^2+^-dependent esterase activity has been reported for TPE from *Meleagris gallopavo* (Cherif and Gargouri, 2010), an esterase from *Anoxybacillus gonensis* (Colak et al., 2005), and EIc and EII from *Cucurbita pepo* (Fahmy et al., 2008). In contrast, Ca^2+^ had a weak influence on the enzyme activity of MtEst45 from *M. thermotolerans* DAU221. Similar results have been reported for EstC from *Streptomyces coelicolor* (Brault et al., 2012), LipG from a metagenomic library of tidal flat sediments (Lee et al., 2006), LipEH166 from an intertidal flat metagenomic library (Kim et al., 2009), LipA3 from *Thermoanaerobacter tengcongensis* (Rao et al., 2011), Est_P1 from a metagenomic library of neritic sediments (Peng et al., 2011), and EstS1 from *Sulfobacillus acidophilus* (Zhang et al., 2014). Zinc ions (Zn^2+^) strongly inhibited the activity of MtEst45. Similar results have been reported for EstF from a deep-sea metagenomic library (Fu et al., 2011), EIc and EII from *Cucurbita pepo* (Fahmy et al., 2008), EstC from *Streptomyces coelicolor* (Brault et al., 2012), LipEH166 from an intertidal flat metagenomic library (Kim et al., 2009), LipA3 from *Thermoanaerobacter tengcongensis* (Rao et al., 2011), Est97 from an arctic intertidal metagenomic library (Fu et al., 2013), TPE from *Meleagris gallopavo* (Cherif and Gargouri, 2010), and Est_P1 from a metagenomic library of neritic sediments (Peng et al., 2011). Copper (Cu^2+^) also strongly inhibited the activity of MtEst45. Similar results have been reported for EstF from a deep-sea metagenomic library (Esteban-Torres et al., 2014), TPE from *Meleagris gallopavo* (Cherif and Gargouri, 2010), EIc and EII from *Cucurbita pepo* (Fahmy et al., 2008), LipEH166 from an intertidal flat metagenomic library (Kim et al., 2009), EstOF4 from *Bacillus* sp. (Rao et al., 2013), LipA3 from *Thermoanaerobacter tengcongensis* (Rao et al., 2011), EstAS from an activated sludge metagenomic library (Zhang et al., 2009), and Est_P1 from a metagenomic library of neritic sediments (Peng et al., 2011). Many lipolytic enzymes contain two or three conserved disulfide bridges, which are important for substrate binding or recognition (Cygler et al., 1993). To determine the presence of a thiol group in MtEst45, the enzyme was incubated with mercuric ions (Hg^2+^) and β-mercaptoethanol. Hg^2+^ completely inhibited enzyme activity. 10 mM β-mercaptoethanol also strongly inhibited enzyme activity. EstF from a deep-sea metagenomic library showed a 65% inhibition of its maximal activity with 5 mM β-mercaptoethanol (Fu et al., 2011). EstOF4 from *Bacillus* sp. was 90% inhibited by 1 mM Hg^2+^ (Rao et al., 2013). DEPC, known as a histidine inhibitor, also demonstrated drastic MtEst45 enzyme activity inhibition. Similar results have been reported for AML from *Amycolatopsis mediterranei* (Dheeman et al., 2011), EstOF4 from *Bacillus* sp. (Rao et al., 2013), and LipA3 from *Thermoanaerobacter tengcongensis* (Rao et al., 2011). These result suggest that the thiol group and histidine residue are present and important for catalytic function. The activity inhibition of MtEst45 by PMSF demonstrates that a serine is involved in the catalytic center because PMSF could mimic the first transition state in ester bond hydrolysis, presumably by covalently linking to the hydroxyl group of a serine in the active site (Das et al., 2000). Most lipolytic enzymes, such as esterase and lipases, have a serine residue at the enzyme active site (Arpigny and Jaeger, 1999). The lipases are poorly inhibited by PMSF because a hydrophobic domain covers the enzyme's active site, making it in-accessible to the reagent (Das et al., 2000; Fojan et al., 2000; Bornscheuer, 2002; Bornscheuer et al., 2002; Cherif and Gargouri, 2010). Similar results have been reported for EstF from a deep-sea metagenomic library (Fu et al., 2011), EstC from *Streptomyces coelicolor* (Brault et al., 2012), AML from *Amycolatopsis mediterranei* (Dheeman et al., 2011), EstOF4 from *Bacillus* sp. (Rao et al., 2013), Est55 and Est30 from *Geobacillus stearothermophilus* (Ewis et al., 2004), Est30 from *Geobacillus kaustophilus* (Montoro-García et al., 2009), and LipA3 from *Thermoanaerobacter tengcongensis* (Rao et al., 2011). Because MtEst45was isolated from marine bacterium, this enzyme was affected by salt concentrations. MtEst45 was stable in a wide range of salt concentrations (Figure 7). In the presence of 0.5 M NaCl, MtEst45 exhibited approximately 75% enzyme activity, as compared to the standard enzyme assay conditions. Similarly, EstHE1 from the marine metagenome library exhibited approximately 75% enzyme activity in 0.5 M NaCl concentrations (Okamura et al., 2010). Although, MtEst45 and EstHE1 had a slight decrease in the high salt concentrations from 0.5 M to 4 M NaCl, the activity of these enzymes was maintained.

In conclusion, a new lipolytic enzyme family has been identified. This family includes MtEst45 from *M. thermotolerans* DAU221, which was isolated from the eastern coast of the Republic of Korea. MtEst45 is the first experimentally characterized enzyme of this new family of bacterial lipolytic enzymes from marine bacterial sources. More detailed enzymatic profiles of MtEst45 were required for the biotechnological applications.

## Author contributions

Conceived, designed, and performed the experiments: YL. Analyzed the data: YL. Contributed reagents/materials/analysis tools: YL. Wrote the paper: YL.

### Conflict of interest statement

The author declares that the research was conducted in the absence of any commercial or financial relationships that could be construed as a potential conflict of interest.
